# Why children differ in motivation to learn: Insights from over 13,000 twins from 6 countries

**DOI:** 10.1016/j.paid.2015.02.006

**Published:** 2015-07

**Authors:** Yulia Kovas, Gabrielle Garon-Carrier, Michel Boivin, Stephen A. Petrill, Robert Plomin, Sergey B. Malykh, Frank Spinath, Kou Murayama, Juko Ando, Olga Y. Bogdanova, Mara Brendgen, Ginette Dionne, Nadine Forget-Dubois, Eduard V. Galajinsky, Juliana Gottschling, Frédéric Guay, Jean-Pascal Lemelin, Jessica A.R. Logan, Shinji Yamagata, Chizuru Shikishima, Birgit Spinath, Lee A. Thompson, Tatiana N. Tikhomirova, Maria G. Tosto, Richard Tremblay, Frank Vitaro

**Affiliations:** a Laboratory for Cognitive Investigations and Behavioral Genetics, Tomsk State University, Russia; b Goldsmiths, University of London, London, UK; c King’s College London, MRC Social, Genetic and Developmental Psychiatry Centre, Institute of Psychiatry, Psychology & Neuroscience, UK; d Psychological Institute, Russian Academy of Education, Moscow, Russia; e Université Laval, Québec, Canada; f The Ohio State University, Columbus, OH, USA; g Department of Psychology, Saarland University, Saarbrücken, Germany; h Department of Psychology, University of California, Los Angeles, USA; i Keio University, Tokyo, Japan; j Université du Québec à Montréal, Montréal, Canada; k Département de psychoéducation, Université de Sherbrooke, Sherbrooke, Canada; l Crane Center for Early Child Research and Policy, The Ohio State University, Columbus, OH, USA; m National Center for University Entrance Examinations, Tokyo, Japan; n Department of Psychology, Heidelberg University, Germany; o Department of Psychological Sciences, Case Western Reserve University, Cleveland, OH, USA; p Université de Montréal, Montréal, Canada; q School of Public Health, Physiotherapy and Public Health, University College Dublin, Dublin, Ireland; r Department of Psychology, University of York, UK; s Tomsk State University, Russia

**Keywords:** Enjoyment, Self-perceived ability, Twin studies, Cross-cultural study, Teacher/classroom effect, Individual differences

## Abstract

Little is known about why people differ in their levels of academic motivation. This study explored the etiology of individual differences in enjoyment and self-perceived ability for several school subjects in nearly 13,000 twins aged 9–16 from 6 countries. The results showed a striking consistency across ages, school subjects, and cultures. Contrary to common belief, enjoyment of learning and children’s perceptions of their competence were no less heritable than cognitive ability. Genetic factors explained approximately 40% of the variance and all of the observed twins’ similarity in academic motivation. Shared environmental factors, such as home or classroom, did not contribute to the twin’s similarity in academic motivation. Environmental influences stemmed entirely from individual specific experiences.

## Highlights

• Genes rather than environment contribute to family resemblance in academic motivation.
• Environmental influences stemmed entirely from individual specific experiences.
• Attending same vs. different classes did not affect twin similarity in motivation.
• Results are similar across ages, countries and academic subjects.

## 1 Introduction

Academic motivation refers to a wide range of traits, such as individuals’ educationally relevant beliefs, perceptions, values, interests, enjoyment, and attitudes (Ryan & Deci, 2000; Urdan & Midgley, 2003; Wigfield & Eccles, 2000) that are associated to school achievement (Elliot & Dweck, 2005). The etiology of individual differences in these traits remains poorly understood. In this paper, we focused on two important motivational constructs: enjoyment of learning (e.g., interest, liking), usually referred to intrinsic motivation; and self-perceived ability, also known as academic self-concept (e.g., children’s perception of how good they are at school subjects).

Several recent studies found self-perceived ability to be substantially heritable (Spinath, Spinath, & Plomin, 2008), even when controlling for general cognitive ability (Greven, Harlaar, Kovas, Chamorro-Premuzic, & Plomin, 2009; Luo, Kovas, Haworth, & Plomin, 2011). In terms of environmental contributions, up to 60% of the variance in enjoyment and self-perceived ability is explained by non-shared experiences (Spinath et al., 2008).

Despite the absence of significant shared environmental effects shown by recent large twin studies, several educational studies found a link between aspects of academic motivation and family/classroom-wide factors, such as classroom climate, peer influence, and mothers’ motivational practices in child’s education (Church, Elliot, & Gable, 2001; Gottfried, Fleming, & Gottfried, 1994; Marsh, Martin, & Cheng, 2008; Ryan, 2000). One possible explanation for this inconsistency is that environmental influences may be correlated with genetic effects (Plomin, DeFries, Knopik, & Neiderhiser, 2012). For example, parental involvement in child’s education may have a causal effect on motivation or/and reflect partly genetically driven parental levels of education, ability, and motivation. Some observed classroom effects might also stem from intake selection (e.g., ability streaming). Most research into the relevant home environmental influences examines only one child per family, which makes it difficult to establish whether the environmental effects operate in a family-wide or child-specific manner. It is possible that even objectively shared experiences, such as availability of educational resources at home, act as child-specific experiences through gene-environment correlation, a mechanism through which children in the same home modify their shared environment into individual experiences.

The role of teachers in shaping children’s academic motivation has been extensively studied (Chirkov & Ryan, 2001; Church et al., 2001; Reeve & Jang, 2006; Urdan & Midgley, 2003). Research suggested that teachers can promote the development of intrinsic motivation (e.g., enjoyment, liking) by encouraging students’ autonomy, providing feedback and optimal challenges, and adopting a caring attitude towards students (Chirkov & Ryan, 2001; Ryan & Deci, 2000). However, teacher effects cannot be easily disentangled from other potential effects of classroom resources, number of children in the class, and teacher unfacilitated classroom-peer interactions (Olson, Keenan, Byrne, & Samuelsson, 2014). Such teacher/classroom effects vary across development, with potentially stronger or persistent effects at the early stages of the formal education when children are facing systematic instruction and academic feedback for the first time (Church et al., 2001; Kovas, Haworth, Dale, & Plomin, 2007; Reeve & Jang, 2006; Urdan & Midgley 2003).

If teachers/classrooms have a strong average effect on children’s liking a particular school subject, we should expect twins in different classes to be on average less similar in their enjoyment of the subject than those in same classes. Findings on academic achievement are mixed: several studies have found small teacher/classroom influences (Byrne et al., 2010; Nye, Konstantopoulos, & Hedges, 2004), whereas other studies did not find any (Kovas et al., 2007), with a recent review suggesting that classroom performance differences should not be viewed as indicators of teacher quality (Olson et al., 2014). It could be that teachers and classrooms have a non-shared, child-specific influence, possibly interacting with children’s genetic and unique environmental background - leading to unique perceptions and reactions in different children.

The goal of this study was to investigate the relative contribution of genetic and environmental factors to individual differences in enjoyment and self-perceived ability as a function of cultural and educational settings. Twins between 9 and 16 years of age from six different countries were evaluated on their enjoyment of learning and the perception of their competence in several academic disciplines. We also compared twin similarity in same versus different classrooms to evaluate teacher/classroom effects. Finally, we tested whether the first formal teacher/classroom affects later class-wide level of enjoyment and self-perceived ability (Church et al., 2001; Kovas et al., 2007; Reeve & Jang, 2006; Urdan & Midgley, 2003).

## 2 Method

### 2.1 Participants

Data of nearly 13,000 identical twins (monozygotic, MZ) and non-identical (dizygotic, DZ) same-sex twins came from six different ongoing twin studies conducted in United Kingdom (Twins Early Development Study – TEDS; Haworth, Davis, & Plomin, 2012), Canada (Quebec Newborn Twin Study – QNTS; Boivin et al., 2013), Japan (Keio Twin Project; Ando et al., 2013), Germany (Twin study on Cognitive ability, Self-reported Motivation and School performance – CoSMoS; Spinath & Wolf, 2006), United States (Western Reserve Reading Project – WRRP; Petrill, Deater-Deckard, Thompson, DeThorne, & Schatschneider, 2006); and Russia (Russian School Twin Registry – RSTR; Kovas et al., 2012). Detailed information on each sample is presented in the Appendix A.1.

### 2.2 Materials

Across all samples, children reported their level of enjoyment and self-perceived ability of different school subjects by completing questionnaires. Self-reported evaluations of enjoyment and self-perceived ability were collected from the UK twins at ages 9, 12 (Luo et al., 2011; Spinath, Spinath, Harlaar, & Plomin, 2006) and 16 (OECD, 2000, 2003, 2006); Canadian twins at ages 10 and 12 (Guay, Marsh, & Boivin, 2003); Japanese twins at ages 10, 11, 12, 13 and 16 (Pintrich & de Groot, 1990); German twins at ages 9, 11 and 13 (Spinath et al., 2008); US twins at age 12 (Harlaar, Deater-Deckard, Thompson, DeThorne, & Petrill, 2011); and Russian twins at age 16 (OECD, 2000, 2003, 2006). Table 1 summarizes the measures and the overall sample size for each twin study. The table indicates maximum number of children in each sample.

Although the measures used across the samples were not identical, they were designed to tap into the same motivational constructs. Convergence of results under these circumstances warrants greater confidence in their generalizability and replicability beyond specific methodological features. Details of each measure are presented in the Appendix A.2.

### 2.3 Procedure

Analyses were conducted on variables corrected for age and sex within each sample. Where data on opposite-sex DZ twins were available (UK, Canada, Japan, and Germany), we ran the analyses twice, including and excluding opposite sex DZ twins - with very similar results.

The information on whether twins and their co-twins were taught in the same or different classes was also available in the UK sample at ages 7 and 9. We tested whether being in different classes for 8 or more months reduces similarity in the level of enjoyment and perceived ability for the two twins by dividing the sample into same versus different class at age 9. The proportions of twins in same vs. different classrooms were very similar for the two zygosity groups: 60% of MZ twins vs. 59% of DZ twins were taught in the same class. In addition, we split the sample at age 9 into the same teacher/class at age 7 to test whether the first teacher or class had a long-lasting class-wide contribution to academic motivation.

## 3 Analyses

### 3.1 Twin analyses

We examined the etiology of enjoyment and self-perceived ability for every subject at every age and in each sample separately by comparing within-pair similarity for MZ and DZ twins. Heritability (A) can be estimated as twice the difference between the MZ and DZ intra-class correlations (ICCs). Shared environmental influences (C) are suggested if DZ twins’ correlation is more than half of the MZ correlation and are computed by subtracting the heritability from the MZ ICC. Shared environment refers to environmental influences that both members of a twin pair experience and that increases the similarity between them. Factors such as socio-economic status, home environment, and school are often thought to contribute to similarities among family members. Non-shared environmental influences (E) are estimated by subtracting MZ twin correlation from 100% (Plomin et al., 2012). The non-shared environment refers to environmental factors that are experienced differently by each twin of a pair and that increase their dissimilarity. Non-shared environmental influences may include individual specific experiences, such as different peers and classmates, differential treatment by their parents and teachers, and differences in twins’ perceptions of such experiences (Kovas et al., 2007). Non-shared environmental estimates also include measurement error.

### 3.2 Classroom heterogeneity model

The effects of classroom on enjoyment and self-perceived ability were investigated by fitting “the classroom heterogeneity model” to the data available from the UK sample. These model-fitting analyses tested whether the differences in estimates of the ACE parameters for twin pairs studying in the same class and twin pairs studying in different classes were statistically significant. The classroom heterogeneity model is similar to the sex-limitation models used to test for quantitative sex differences (Kovas et al., 2007). To test for the long-lasting (spill-over) effects of the first teacher/classroom experiences on later academic motivation, we performed the same analyses splitting the sample at age 9 into twins who were attending the same versus different classroom when they were 7.

## 4 Results

A wide variation in academic motivation scores was observed within each sample. The data for most measures were normally distributed. Prior to all analyses, where data did not meet the criterion of normality, the necessary transformations were applied (e.g., Vander Waerden, reflect and log). Repeated analyses using transformed and untransformed scores yielded similar results.

### 4.1 Phenotypic correlations

Pearson correlations between enjoyment and self-perceived ability were performed on one twin randomly selected out of each pair, and conducted on scores adjusted for age. Correlations were moderate to strong in all samples: *r* = .41–.79, averaged = .65 (see Table B.1 in the Appendix).

### 4.2 Effects of sex and zygosity

Analyses of variance (ANOVA) were performed within each sample in order to assess the effects of sex and zygosity and their interaction on each variable. The results were adjusted for exact age within each sample. Means, standard deviations and the results of ANOVAs are presented in Appendix (see Tables B.2 and B.3). Overall, boys reported higher perceived ability, with 6 out of 16 comparisons reaching significance (*p* < .05), and higher enjoyment of mathematics and science in all samples, with 5 out of 16 comparisons reaching significance (*p* < .05). The effect size of these differences was small, ranging from less than 1–6% for self-perceived ability, and ranging from less than 1–9% for enjoyment.

On the contrary, girls reported higher perceived ability, with 3 out of 8 significant comparisons (*p* < .05), and with less than 2% of the variance explained by sex. They also reported higher enjoyment of reading and language academic subjects, with 5 out of 8 significant comparisons (*p* < .05). Between 1% and 5% of the variance in enjoyment was explained by sex. Overall, MZ and DZ twins showed similar levels of enjoyment and self-perceived ability within each sample.

In the UK sample, we were also able to test for a main effect of zygosity, class, and zygosity by class interaction on enjoyment and self-perceived ability. In other words, we tested whether twins showed greater enjoyment and higher self-perceived ability when they were taught in the same (as opposed to different) classroom; and whether this effect was specific (or more prominent) to one of the zygosity groups. We conducted a series of 2 by 2 ANOVAs, for each school subject, with zygosity (MZ vs. DZ) and class (same vs. different) – as two factors. For enjoyment, we found no class or zygosity effect and no interaction. In other words, average levels of enjoyment of the subjects were similar for MZ and DZ twin groups, irrespective of whether they attended the same or different classes. For self-perceived ability, we found no zygosity effect but a significant effect of class on self-perceived ability for English and Maths: children in the same classroom showed a slightly higher level of self-perceived ability. However, this effect was negligible, explaining less than 1% of the variance. No significant interactions were found. These results suggest that twins (both MZ and DZ) have slightly higher self-perceived ability when taught in the same (rather than different) class. However, in this study, this effect was too weak to justify any further interpretation.

### 4.3 Heritability of enjoyment and self-perceived ability

MZ and DZ ICCs are presented in Tables 2 and 3, separately for enjoyment and self-perceived ability. Striking similarities were observed across the ages, school subjects and samples for both enjoyment (average MZ ICC = .46; average DZ ICC = .16) and self-perceived ability (average MZ ICC = .46; average DZ ICC = .19).

**Fig. C1:**
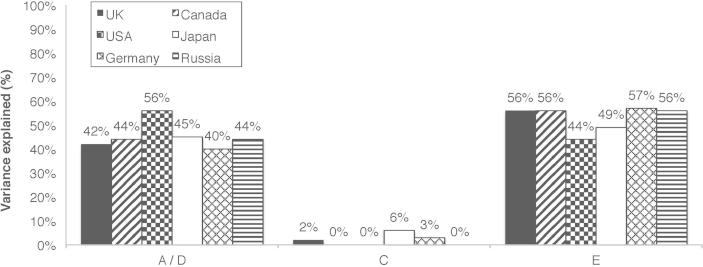
Relative contributions of genetic (A/D), shared (C) and non-shared (E) environmental factors to individual differences in academic motivational traits by country, averaged across age, school subject and construct. Enjoyment of German language at age 9 was excluded from the figure as this sample produced anomalous result of absence of genetic effects.

**Fig. 1:**
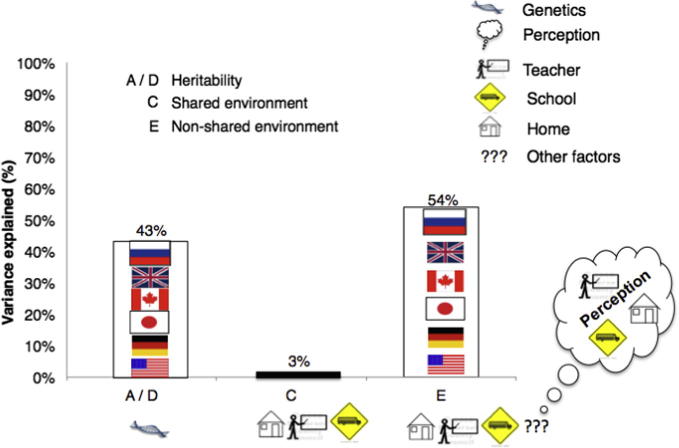
Average heritability, shared environment and non-shared environment for enjoyment and self-perceived ability from 6 large-twins samples. The sample in which anomalous result of non-significant heritability was found (enjoyment of German at age 9) was excluded from the figure. The schematic drawing of home, teacher and school are included in both shared and non-shared environment legend – to reflect that such factors can lead to similarities and differences in family members. Non-shared environments may also include perceptions of these factors.

Overall, the results showed that individual differences in enjoyment and self-perceived ability are explained to a similar extent by genetic and individual-specific (i.e., non-shared) environmental factors. Genetic contributions ranged from 16% to 69% across the samples; non-shared environmental contributions ranged from 31% to 75%. Some potentially meaningful cultural specific and subject specific effects were observed. For example, modest shared environmental influences were found for enjoyment and self-perceived ability in German-language at age 9, and for self-perceived ability at age 13; modest but significant shared environmental influences (10%) were found for self-perceived ability in science at age 9; and moderate shared environment was found in the Japanese sample for self-perceived ability in mathematics at age 11. Although these four exceptions, no significant shared environmental influences on these constructs were found. Figure 1 presents the results averaged across age, school subject, motivational construct, and country (see Fig. C.1 in the Appendix for the results split by country).

### 4.4 Classroom effect on enjoyment and self-perceived ability

Children in different classes did not rate their enjoyment of the subjects or their self-perceived ability less similar than those in same classes, with equal genetic (A), shared (C) and non-shared environmental (E) estimates for the two groups. We also tested the assumption that the effect of the first formal teacher may have a continuous or delayed effect on later motivational levels by splitting the sample at 9 years of age by whether the children were taught by the same or different teacher at age 7. The ACE parameters could be equated when the UK sample was split by whether the twins attended the same versus different classes at age 7. In other words, no class-wide effect on contemporaneous or later levels of enjoyment and self-perceived ability was found (see Tables B.4–B.9 in the Appendix).

## 5 Discussion

Overall, the pattern of results for enjoyment and self-perceived ability was highly similar, which is not surprising as these constructs were moderately to strongly correlated for each school subject in each sample. The results showed high consistency across ages, school subjects and cultures in the etiology of individual differences in enjoyment and self-perceived ability. This consistency is particularly striking given the vast cross-cultural differences in schooling and the educational systems involved. The familial similarity in levels of academic motivation was only moderate, even for genetically identical children raised in the same home. With few exceptions, neither enjoyment nor self-perceived ability were influenced by shared environment. In other words, similarities in enjoyment and self-perceived ability in twins growing up in the same family and attending the same schools were entirely explained by their genetic, rather than their environmental relatedness.

However, genetic effects on enjoyment and self-perceived ability varied substantially across the samples. These differences in heritability could reflect some cultural aspects that lead to differences in amount of observed variation explained by genetic factors. The observed differences could also be explained by differences in sample sizes and associated statistical power.

Moreover, attending different classrooms did not increase dissimilarity between twins in their levels of enjoyment and self-perception of competence. Equal similarity between twins attending same and different classrooms cannot be explained with equalising effect of the shared home environment as no such effect was found in this study. These results suggest that similarity in academic motivation for any unrelated individuals stems from their chance genetic similarity, as well as similar individual-specific environmental experiences, rather than similar family/class-wide experiences. Whatever the environmental influences on the levels of enjoyment and self-perceived ability are, they seem to act in a non-shared, individual-specific way, potentially interacting with genetic make-up, experiences and perceptions. Multiple individual-specific life-events, such as birth complications, missing school due to illness, and peer-relations, may contribute to motivation. Effects of family members, teachers, classes, and schools may also be non-shared: parents, siblings, and teachers may actually treat children in the same family/class differently, responding to their individual characteristics (Babad, 1993; Harris & Morgan, 1991; Spengler, Gottschling, & Spinath, 2012). On the other hand, children may perceive their parents, teachers, classmates, and schools differently (Zhou, Lam, & Chang, 2012) – depending on other non-shared environmental and genetic effects. In addition, genetic effects may differ as a function of environment. For example, research suggested that heritability of reading might be moderated by teacher quality or SES status (Taylor, Roehrig, Hensler, Connor, & Schatschneider, 2010).

## 6 Conclusions

Considering the striking consistency of these results across different aspects of academic motivation, different subjects, different ages, and different cultures, we believe that it is time to move away from solely environmental explanations, such as “good” or “bad” home, teacher, and school, for differences in enjoyment and self-perceived ability (Olson et al., 2014). The results convincingly show that, contrary to common belief, enjoyment of learning and children’s perceptions of their competence are no less heritable than cognitive ability (Greven et al., 2009). Surprisingly, unlike cognitive ability, for which shared environment makes a small to moderate contribution across the school years (Petrill et al., 2004), no such contribution was found for these motivational constructs.

It remains unclear to what extent the genetic and non-shared environmental factors contributing to variation in enjoyment and self-perceived ability also contribute to variation in achievement and intelligence (Gottfried et al., 1994). Academic motivation is not independent of achievement, as it develops partly through feedback on performance and in turn may influence achievement (Guay et al., 2003). For example, some studies found bidirectional effects between motivation and performance (Luo et al., 2011).

This and other genetically sensitive studies call for caution when developing interventions aimed at raising academic motivation before more is known about specific mechanisms underlying its variation (Olson et al., 2014). Current educational policies are based on average effects and are designed to operate at the family-wide and class-wide levels. However, the present research suggests that many true effects may be masked within any class or home, and that individual-specific educational approaches are required.

## Figures and Tables

**Table B.1:** Phenotypic correlations between enjoyment and self-perceived ability at each age and for each school subject in each sample.

Age	Country	School subject	*r*	*p*	*N*
9 years	UK	Math	.73	.01	2276
		English	.57	.01	2272
		Science	.64	.01	2263
	Germany	Math	.70	.00	261
		German	.65	.00	261

10 years	Canada (Quebec)	Math	.69	.00	272
		Reading	.41	.00	272
	Japan	Math	.65	.00	181

11 years	Germany	Math	.79	.00	261
		German	.76	.00	261
	Japan	Math	.63	.00	172

12 years	UK	Math	.73	.01	3854
		English	.61	.01	3841
		Science	.67	.01	3835
	Canada (Quebec)	Math	.66	.00	262
		Reading	.59	.00	262
	Japan	Math	.72	.00	179
	USA	Reading	.60	.00	361

13 years	Germany	Math	.76	.00	261
		German	.45	.00	261
	Japan	Math	.66	.00	121

16 years	UK	Math	.53	.01	1653
	Japan	Math	.75	.00	95
	Russia	Math	.55	.01	51

**Table B.2:** Self-perceived ABILITY: means (SD) for ages 9–16, by zygosity and sex; and ANOVA results showing significance and effect size.

Enjoyment	Countries	School subjects	Zygosity		Sex		ANOVA					
			MZ	DZss	Female	Male	Zygosity		Sex		Zygosity * sex	
							*p*	*η*^2^	*p*	*η*^2^	*p*	*η*^2^
9 years	UK	Math	−.07 (1.02)	.00 (1.01)	−.18 (.98)	.20 (1.01)	**.01**	.00	**.00**	.03	**.02**	.00
		English	.02 (1.01)	−.01 (.99)	.17 (.95)	−.17 (1.04)	.58	.00	**.00**	.03	**.02**	.00
		Science	.00 (1.00)	−.01 (.98)	−.05 (.98)	.07 (1.01)	.74	.00	**.00**	.00	.14	.00
	Germany	Math	.05 (.98)	−.14 (.98)	−.17 (1.01)	.08 (.95)	.37	.01	.25	.01	.49	.01
		German	.12 (.88)	−.02 (.99)	.20 (.81)	−.13 (1.03)	**.04**	.02	.**04**	.02	.35	.01

10 years	Canada (Quebec)	Math	.03 (1.03)	−.04 (.95)	−.03 (1.07)	.03 (.92)	.58	.00	.67	.00	.73	.00
		Reading	.07 (1.01)	−.10 (.98)	.05 (.99)	−.05 (1.02)	.17	.01	.40	.00	.75	.00
	Japan	Math	.11 (1.04)	−.16 (1.01)	−.10 (1.05)	.12 (1.00)	.10	.02	.35	.01	.33	.01

11 years	Germany	Math	−.01 (.95)	−.12 (.98)	−.26 (.99)	.14 (.90)	.43	.01	.22	.01	**.05**	.02
		German	.01 (.92)	−.02 (.98)	.15 (.94)	−.17 (.96)	.83	.00	.66	.00	.44	.01
	Japan	Math	.02 (1.04)	−.03 (.97)	−.15 (1.06)	.22 (.91)	.61	.00	.01	.04	.21	.01

12 years	UK	Math	−.01 (.99)	−.02 (.99)	−.08 (.97)	.10 (1.03)	.29	.00	.46	.00	**.00**	.01
		English	.00 (.99)	.01 (.99)	.24 (.96)	−.24 (.98)	.35	.00	**.00**	.05	.17	.01
		Science	−.03 (1.00)	.01 (.99)	−.02 (.98)	.03 (1.01)	.09	.00	.06	.00	.42	.00
	Canada (Quebec)	Math	.06 (1.00)	−.07 (1.01)	−.02 (1.01)	.02 (.99)	.27	.01	.78	.00	.36	.00
		Reading	−.02 (.96)	.02 (1.05)	.06 (.98)	−.06 (1.03)	.74	.00	.22	.01	.07	.01
	Japan	Math	−.07 (1.11)	.14 (.95)	−.05 (1.06)	.10 (1.07)	.55	.00	.77	.00	.08	.02
	USA	Reading	.04 (.63)	−.03 (.68)	.07 (.65)	−.09 (.68)	.20	.00	**.04**	.01	.26	.00

13 years	Germany	Math	.08 (.89)	−.11 (1.04)	−.20 (.94)	.16 (.97)	.26	.01	.21	.01	.55	.01
		German	.16 (.86)	.03 (1.04)	.21 (.95)	−.07 (.99)	**.00**	.04	**.02**	.02	.30	.01
	Japan	Math	.12 (1.00)	−.36 (.82)	−.28 (.93)	.37 (.92)	**.01**	.06	**.00**	.09	.68	.00

16 years	UK	Math	.02 (.99)	.03 (.99)	−.09 (1.03)	.14 (.94)	.26	.00	**.01**	.06	.11	.00
	Japan	Math	−.04 (.98)	.02 (1.04)	−.15 (1.02)	.19 (1.00)	.54	.00	.09	.03	.72	.00
	Russia	Math	.08 (1.14)	−.02 (.81)	−.19 (1.06)	.19 (.97)	.33	.01	.16	.02	.66	.02

*Note.* The results were adjusted for exact age within each sample. DZss = same sex DZ twins. Significant differences in zygosity, sex and sex * zygosity interaction are indicated in bold.

**Table B.3:** Enjoyment: means (SD) for ages 9–16, by zygosity and sex; and ANOVA results showing significance and effect size.

Self- perceived ability (SPA)	Countries	School subjects	Zygosity		Sex		ANOVA					
			MZ	DZss	Female	Male	Zygosity		Sex		Zygosity * sex	
							*p*	*η*^2^	*p*	*η*^2^	*p*	*η*^2^
9 years	UK	Math	-.05 (.99)	.01 (1.02)	−.19 (.99)	.22 (.99)	.09	.00	**.00**	.04	**.01**	.00
		English	.02 (1.00)	.04 (1.00)	.10 (.96)	−.09 (1.03)	.50	.00	**.01**	.00	**.00**	.01
		Science	.02 (.98)	.00 (.99)	−.04 (.97)	.07 (1.00)	.75	.00	**.00**	.00	**.02**	.00
	Germany	Math	.07 (.99)	−.18 (1.05)	−.14 (1.00)	.04 (1.04)	.15	.02	.37	.00	.36	.01
		German	.07 (.91)	−.02 (.99)	.14 (.93)	−.12 (.98)	.09	.02	.11	.01	.54	.01

10 years	Canada (Quebec)	Math	.00 (.99)	−.01 (1.02)	−.11 (1.00)	.12 (.99)	.94	.00	.06	.01	.96	.00
		Reading	−.01 (.98)	.02 (1.03)	.01 (.98)	−.01 (1.03)	.83	.00	.78	.00	.60	.00
	Japan	Math	.05 (.81)	−.06 (.79)	−.06 (.79)	.08 (.81)	.47	.00	.31	.01	.69	.00

11 years	Germany	Math	−.03(1.02)	−.09 (.95)	−.20 (.99)	.13 (.95)	.23	.01	.23	.01	.94	.00
		German	.00 (.88)	−.04(1.04)	.09 (1.03)	−.14 (.93)	.59	.00	.46	.00	.84	.00
	Japan	Math	.01 (.83)	−.01 (.73)	−.01 (.78)	.01 (.81)	.96	.00	.71	.00	.36	.00

12 years	UK	Math	−.02 (.99)	−.01 (.99)	−.16 (.96)	.18 (1.01)	.40	.00	**.00**	.03	**.00**	.00
		English	.00 (1.00)	.02 (.95)	.14 (.96)	−.14 (.98)	.53	.00	**.01**	.02	.15	.00
		Science	.00 (.99)	−.01 (.98)	−.01 (.98)	.02 (1.00)	.55	.00	.12	.00	.14	.00
	Canada (Quebec)	Math	.06 (.96)	−.08 (1.05)	−.17 (.95)	.19 (1.02)	.16	.01	**.00**	.04	.68	.00
		Reading	.00 (.98)	.00 (1.03)	.01 (.98)	−.01 (1.02)	.95	.00	.82	.00	.66	.00
	Japan	Math	−.05 (.91)	.10 (.86)	−.06 (.85)	.11 (.97)	.53	.00	.49	.00	.19	.01
	USA	Reading	−.00 (.72)	.00 (.63)	.08 (.67)	−.11 (.69)	.93	.00	**.01**	.02	.63	.00
		General School	.12 (3.06)	−.14 (3.21)	−.17 (3.02)	.23 (3.03)	.40	.00	**.04**	.01	.31	.00

13 years	Germany	Math	−.02 (.96)	−.09 (1.09)	−.25 (1.03)	.14 (1.01)	.75	.00	.29	.00	.71	.00
		German	.00 (.88)	−.04 (1.04)	.09 (1.03)	−.14 (.93)	.59	.00	.46	.00	.84	.00
	Japan	Math	.04 (.83)	−.12 (.83)	−.16 (.81)	.21 (.77)	.27	.01	**.02**	.05	.89	.00

16 years	UK	Math	.00 (.97)	.03 (.95)	−.17 (.99)	.30 (.85)	.93	.00	**.00**	.06	.64	.00
	Japan	Math	−.02 (.88)	.05 (.82)	−.18 (.87)	.22 (.80)	.98	.00	.08	.03	.45	.01
	Russia	Math	−.13 (1.07)	.14 (.87)	.00 (.94)	.11 (1.06)	.82	.01	.27	.04	.96	.00

*Note.* The results were adjusted for exact age within each sample. DZss = same sex DZ twins. Significant differences in zygosity, sex and sex * zygosity interaction are indicated in bold.

**Table B.4:** Intra-class correlations of enjoyment and self-perceived ability at age 9 for same- vs. different-teacher/class at age 9; & genetic (A), shared environmental (C) and non-shared environmental (E) estimates for the whole sample.

TEDS		MZ pairs		DZss pairs		A	C	E
9 years		Same teacher	Different teacher	Same teacher	Different teacher			
English	Enjoyment	.42 (*N* = 670)	.40 (*N* = 453)	.19 (*N* = 1121)	.12 (*N* = 773)	.40 (.34-.44)	.00 (.00-.04)	.60 (.56-.65)
	Self-perceived ability	.41 (*N* = 677)	.40 (*N* = 452)	.14 (*N* = 1131)	.14 (*N* = 776)	.38 (.32-.42)	.00 (.00-.03)	.62 (.58-.67)

Math	Enjoyment	.39 (*N* = 666)	.38 (*N* = 452)	.14 (*N* = 1121)	.11 (*N* = 772)	.35 (.29-.39)	.00 (.00-.04)	.65 (.61-.69)
	Self-perceived ability	.42 (*N* = 675)	.39 (*N* = 453)	.10 (*N* = 1127)	.06 (*N* = 776)	.35 (.31-.40)	.00 (.00-.02)	.65 (.30-.69)

Science	Enjoyment	.35 (*N* = 662)	.34 (*N* = 449)	.20 (*N* = 1119)	.14 (*N* = 769)	.31 (.18-.38)	.03 (.00-.13)	.66 (.62-.72)
	Self-perceived ability	.37 (*N* = 671)	.35 (*N* = 455)	.17 (*N* = 1125)	.19 (*N* = 776)	.36 (.23-.40)	.00 (.00-.10)	.64 (.60-.69)

*Note.* DZss = same sex DZ twins. 60% of twins had the same teacher/classroom at age 9. As suggested by the MZ and DZ correlations, the twins at age 9 were no more similar in their enjoyment and perceived ability when they were taught by the same teacher in the same class or by different teacher in different classes at age 9.

**Table B.5:** Fit statistics of the teacher/class heterogeneity-homogeneity models for enjoyment and self-perceived ability at age 9 for same- vs. different-teacher/class twin pairs at age 9.

		6 parameters model		3 parameters model		Homogeneity model
		AIC	BIC	AIC	BIC	*χ*^2^
English	Enjoyment	4866.354	−41027.20	4865.178	−41050.85	*χ*^2 ^> .19
	Self-perceived ability	4888.890	−41161.99	4883.591	−41189.76	*χ*^2 ^> .87

Maths	Enjoyment	4939.787	−40893.83	4934.389	−40921.70	*χ*^2 ^> .89
	Self-perceived ability	4968.001	−41045.42	4962.815	−41073.08	*χ*^2 ^> .85

Science	Enjoyment	4939.577	−40804.14	4937.640	−40828.56	*χ*^2 ^> .25
	Self-perceived ability	4958.279	−41017.68	4952.657	−41045.78	*χ*^2 ^> .94

*Note.* The best fitting model equated the ACE parameters for same- and different-teacher/class (Homogeneity model).

**Table B.6:** ACE components of enjoyment and self-perceived ability at age 9 for same- vs. different-teacher/class at age 9.

		ACE: same teacher/class			ACE: different teacher/class		
		A	C	E	A	C	E
English	Enjoyment	.41 (.30–.47)	.00 (.00–.08)	.59 (.53–.64)	.37 (.28–.44)	.00 (.00–.06)	.63 (.56–.70)
	Self-perceived ability	.38 (.30–.43)	.00 (.00–.05)	.62 (.57–.68)	.38 (.27–.45)	.00 (.00–.07)	.62 (.55–.69)

Maths	Enjoyment	.36 (.27–.42)	.00 (.00–.06)	.64 (.58–.69)	.33 (.23–.40)	.00 (.00–.07)	.67 (.60–.74)
	Self–perceived ability	.37 (.31–.43)	.00 (.00–.03)	.63 (.57–.69)	.33 (.25–.40)	.00 (.00–.04)	.67 (.60–.75)

Science	Enjoyment	.25 (.08–.39)	.08 (.00–.21)	.66 (.60–.73)	.32 (.16–.39)	.00 (.00–.12)	.68 (.61–.75)
	Self-perceived ability	.36 (.21–.41)	.00 (.00–.11)	.64 (.59–.70)	.32 (.11–.42)	.03 (.00–.18)	.65 (.58–.73)

*Note.* ACE parameters estimated separately for twin pairs in the same- vs. different- teacher/class at age 9. As reported in the main report, the parameters could be equated across the groups.

**Table B.7:** Intra-class correlations for enjoyment and self-perceived ability at age 9 for same- vs. different-teacher/class at age 7; & genetic (A), shared environmental (C) and non-shared environmental (E) estimates for the whole sample.

TEDS		MZ pairs		DZss pairs		A	C	E
9 years		Same teacher	Different teacher	Same teacher	Different teacher			
English	Enjoyment	.43 (*N* = 680)	.41 (*N* = 362)	.19 (*N* = 1126)	.10 (*N* = 613)	.40 (.35–.45)	.00 (.00–.04)	.60 (.55–.64)
	Self-perceived ability	.40 (*N* = 684)	.44 (*N* = 364)	.16 (*N* = 1137)	.14 (*N* = 616)	.39 (.33–.43)	.00 (.00–.00)	.61 (.57–.65)

Math	Enjoyment	.40 (*N* = 676)	.35 (*N* = 361)	.10 (*N* = 1126)	.16 (*N* = 612)	.35 (.29–.39)	.00 (.00–.04)	.65 (.61–.70)
	Self-perceived ability	.41 (*N* = 683)	.38 (*N* = 364)	.08 (*N* = 1133)	.11 (*N* = 616)	.35 (.30–.40)	.00 (.00–.02)	.65 (.60–.70)

Science	Enjoyment	.37 (*N* = 672)	.30 (*N* = 358)	.17 (*N* = 1125)	.21 (*N* = 610)	.29 (.15–.38)	.04 (.00–.15)	.66 (.62–.72)
	Self-perceived ability	.37 (*N* = 612)	.34 (*N* = 366)	.17 (*N* = 1133)	.21 (*N* = 615)	.33 (.20–.40)	.02 (.00–.12)	.65 (.60–.70)

*Note.* DZss = same sex DZ twins. 65% of twins had the same teacher/classroom at age 7. As suggested by the MZ and DZ correlations, the twins at age 9 were no more similar in their enjoyment and perceived ability when they were taught by the same teacher in the same class or by different teacher in different classes at age 7.

**Table B.8:** Fit statistics of the teacher/class heterogeneity-homogeneity models for enjoyment and self-perceived ability at age 9 for same- and different-teacher/class twin pairs at age 7.

		6 parameters model		3 parameters model		Homogeneity model
		AIC	BIC	AIC	BIC	*χ*^2^
English	Enjoyment	4461.512	−36239.66	4457.283	−36265.50	*χ*^2 ^> .62
	Self-perceived ability	4477.159	−36382.44	4472.444	−36408.76	*χ*^2 ^> .73

Maths	Enjoyment	4540.976	−36102.59	4535.062	−36130.11	*χ*^2 ^> .99
	Self-perceived ability	4566.503	−36257.09	4560.706	−36284.49	*χ*^2 ^> .98

Science	Enjoyment	4551.606	−36019.95	4547.742	−36045.41	*χ*^2 ^> .54
	Self-perceived ability	4518.871	−36283.12	4514.690	−36308.91	*χ*^2 ^> .61

*Note.* The best fitting model equated the ACE parameters for same- and different-teacher/class (Homogeneity model).

**Table B.9:** ACE components of enjoyment and self-perceived ability at age 9 for same- vs. different-teacher/class at age 7.

		ACE: same teacher/class			ACE: different teacher/class		
		A	C	E	A	C	E
English	Enjoyment	.42 (.31–.48)	.00 (.00–.08)	.58 (.52–.63)	.36 (.27–.44)	.00 (.00–.06)	.64 (.56–.72)
	Self-perceived ability	.39 (.29–.44)	.00 (.00–.06)	.61 (.56–.67)	.40 (.29–.47)	.00 (.00–.08)	.60 (.53–.68)

Maths	Enjoyment	.35 (.28–.41)	.00 (.00–.04)	.65 (.59–.71)	.34 (.12–.41)	.00 (.00–.17)	.66 (.59–.75)
	Self-perceived ability	.35 (.29–.41)	.00 (.00–.03)	.65 (.59–.71)	.35 (.24–.43)	.00 (.00–.07)	.65 (.57–.73)

Science	Enjoyment	.36 (.19–.41)	.00 (.00–.12)	.64 (.59–.71)	.16 (.00–.37)	.13 (.00–.29)	.71 (.62–.80)
	Self-perceived ability	.33 (.20–.40)	.02 (.00–.12)	.65 (.60–.70)	.32 (.11–.42)	.03 (.00–.18)	.65 (.58–.73)

*Note.* ACE parameters estimated separately for twin pairs in the same- vs. different-teacher/class at age 7. As reported in the main report, the parameters could be equated across the groups.

**Table 1:** Sample size in each country by age and concepts of enjoyment and self-perceived ability (SPA).

	UK		Canada		Japan		Germany		USA		Russia	
	Enjoy	SPA	Enjoy	SPA	Enjoy	SPA	Enjoy	SPA	Enjoy	SPA	Enjoy	SPA
9 years	2285	2294					508	508				
10 years			529	529	366	369						
11 years					346	348	508	508				
12 years	3855	3855	516	516	366	360			363	361		
13 years					242	243	508	508				
16 years	1667	1667			193	193					74	74

**Table 2:** Enjoyment: twin correlations and ACE parameters.

Enjoyment	Country	School subject	MZ	DZss	A/D	C	E
9 years	UK	Math	.38 (*N* = 1192)	.14 (*N* = 1049)	.38	.00	.62
		English	.41 (*N* = 1197)	.22 (*N* = 1051)	.38	.03	.59
		Science	.33 (*N* = 1185)	.18 (*N* = 1047)	.30	.03	.67
	Germany	Math	.38 (*N* = 133)	.20 (*N* = 121)	.36	.02	.62
		German	.31 (*N* = 133)	.29 (*N* = 121)	.04	.27	.69

10 years	Canada (Québec)	Math	.34 (*N* = 153)	−.14^a^ (*N* = 108)	.34	.00	.66
		Reading	.46 (*N* = 153)	.02^a^ (*N* = 108)	.46	.00	.54
	Japan	Math	.50 (*N* = 109)	.21^a^ (*N* = 77)	.50	.00	.50

11 years	Germany	Math	.48 (*N* = 133)	.24 (*N* = 121)	.48	.00	.52
		German	.60 (*N* = 133)	.30 (*N* = 121)	.60	.00	.40
	Japan	Math	.50 (*N* = 107)	.28 (*N* = 68)	.44	.06	.50

12 years	UK	Math	.46 (*N* = 2020)	.20 (*N* = 1823)	.46	.00	.54
		English	.48 (*N* = 2020)	.20 (*N* = 1817)	.48	.00	.52
		Science	.43 (*N* = 2016)	.22 (*N* = 1816)	.42	.01	.57
	Canada (Québec)	Math	.42 (*N* = 147)	−.01^a^ (*N* = 111)	.42	.00	.58
		Reading	.59 (*N* = 147)	.27 (*N* = 111)	.59	.00	.41
	Japan	Math	.48 (*N* = 125)	−.03^a^ (*N* = 62)	.48	.00	.52
	USA	Reading	.63 (*N* = 146)	.15 (*N* = 202)	.63	.00	.37

13 years	Germany	Math	.42 (*N* = 133)	.16^a^ (*N* = 121)	.42	.00	.58
		German	.49 (*N* = 133)	.04^a^ (*N* = 121)	.49	.00	.51
	Japan	Math	.53 (*N* = 91)	−.03^a^ (*N* = 31)	.53	.00	.47

16 years	UK	Math	.42 (*N* = 817)	.21 (*N* = 710)	.42	.00	.58
	Japan	Math	.58 (*N* = 68)	.31^a^ (*N* = 32)	.58	.00	.42
	Russia	Math	.41 (*N* = 34)	.15^a^ (*N* = 34)	.41	.00	.59

*Note.* MZ intra-class correlations (ICCs), same-sex DZ (DZss) ICCs, and genetic (A), shared environmental (C) and non-shared environmental (E) estimates. The results were controlled for age and sex. ACE estimates were derived from ICCs using Falconer’s formula. A/D = where DZ ICC is less than half of that of MZ ICC, non-additive genetic (D) effects are implied; in these cases A and D effects cannot be disentangled.

a DZ ICC did not reach significance.

**Table 3:** Self-perceived ability (SPA): Twin correlations and ACE parameters.

SPA	Countries	School subject	MZ	DZss	A/D	C	E
9 years	UK	Math	.40 (*N* = 1204)	.11 (*N* = 1062)	.40	.00	.60
		English	.41 (*N* = 1202)	.16 (*N* = 1023)	.41	.00	.59
		Science	.36 (*N* = 1197)	.23 (*N* = 1053)	.26	.10	.64
	Germany	Math	.39 (*N* = 133)	.24 (*N* = 121)	.30	.09	.61
		German	.55 (*N* = 133)	.35 (*N* = 121)	.40	.15	.45

10 years	Canada (Québec)	Math	.40 (*N* = 153)	-.01^a^ (*N* = 108)	.40	.00	.60
		Reading	.41 (*N* = 153)	.03^a^ (*N* = 108)	.41	.00	.59
	Japan	Math	.25 (*N* = 109)	.16^a^ (*N* = 77)	.18	.07	.75

11 years	Germany	Math	.42 (*N* = 133)	.23 (*N* = 121)	.38	.04	.58
		German	.41 (*N* = 133)	.23 (*N* = 121)	.36	.05	.59
	Japan	Math	.66 (*N* = 107)	.58 (*N* = 68)	.16	.50	.34

12 years	UK	Math	.49 (*N* = 2011)	.15 (*N* = 1813)	.49	.00	.51
		English	.56 (*N* = 2013)	.21 (*N* = 1814)	.56	.00	.44
		Science	.45 (*N* = 2004)	.26 (*N* = 1814)	.38	.07	.55
	Canada (Québec)	Math	.42 (*N* = 147)	.00^a^ (*N* = 111)	.42	.00	.58
		Reading	.48 (*N* = 147)	.01^a^ (*N* = 111)	.48	.00	.52
	Japan	Math	.69 (*N* = 125)	.14^a^ (*N* = 62)	.69	.00	.31
	USA	Reading	.63 (*N* = 144)	.09^a^ (*N* = 207)	.63	.00	.37
		General school	.43 (*N* = 148)	.17 (*N* = 204)	.43	.00	.57

13 years	Germany	Math	.34 (*N* = 133)	.03^a^ (*N* = 121)	.34	.00	.66
		German	.37 (*N* = 133)	.26 (*N* = 121)	.22	.15	.63
	Japan	Math	.49 (*N* = 91)	.35^a^ (*N* = 31)	.49	.00	.51

16 years	UK	Math	.57 (*N* = 811)	.28 (*N* = 803)	.57	.00	.43
	Japan	Math	.47 (*N* = 68)	.24^a^ (*N* = 32)	.47	.00	.53
	Russia	Math	.46 (*N* = 34)	.31^a^ (*N* = 34)	.46	.00	.54

*Note.* MZ intra-class correlations (ICCs), same-sex DZ (DZss) ICCs, and genetic (A), shared environmental (C) and non-shared environmental (E) estimates. The results were controlled for age and sex. ACE estimates were derived from ICCs using Falconer’s formula. A/D = where DZ ICC is less than half of that of MZ ICC, non-additive genetic (D) effects are implied; in these cases A and D effects cannot be disentangled.

a DZ ICC did not reach significance.

## Appendix A.1

### United Kingdom

The Twins’ Early Development Study (TEDS) is a longitudinal study involving a representative sample of twins born in England and Wales in 1994, 1995, and 1996. Families of twins (*N* = 25815) were identified by the UK Office for National Statistics (ONS) from birth records. They were contacted after screening for infant mortality and 16810 families acknowledged their interest in taking part in the study. The first contact general demographic information, including zygosity and information about pregnancy and birth, was collected when the twins were about 18 months. Zygosity was assigned using a parent-reported questionnaire of physical similarity, which is over 95% accurate when compared to DNA testing (Price et al., 2000). For cases where zygosity was unclear, DNA testing was conducted. 12054 families have been involved in TEDS at least for one assessment point. DNA has been collected for more than 7000 pairs. Genome-wide genotyping data for two million DNA markers are available for 3500 individuals. Since enrolment, the families have been invited to take part in studies when the twins were 2, 3, 4, 7, 8, 9, 10, 12, 14, 16 and 18 years of age.

### Canada

The Quebec Newborn Twin Registry was established from all twin births occurring in the Province of Quebec between 1 April 1995 and 31 December 1998. All parents living in the Greater Montreal Area were asked to enroll with their twins in the Quebec Newborn Twin Study (QNTS). A total of 989 families were contacted, of which 672 agreed to participate (68%). Parents were contacted by letter and by phone; laboratory appointments were scheduled for when the twins were five months old (corrected for gestational duration). During the 4–5-h morning laboratory visit, the mother and her twins were assessed on a number of psychophysiological, cognitive and behavioral measures. Two weeks later, the families were also visited at home, where the mother was interviewed and both parents filled out questionnaires. These families were seen in the laboratory and in their home between June 1996 and November 1998. The assessments were done in French or English according to the language of the respondent. A broad range of social, demographic, health, and behavioral data were obtained. Zygosity was ascertained by assessment of physical similarity of twins through aggregation of independent tester ratings using the short version of the Zygosity Questionnaire for Young Twins (Goldsmith, 1991). In addition, DNA was extracted through mouth swabs collected by mothers for 31.3% of the pairs selected at random. DNA-based zygosity was determined using 8–10 polymorphic micro-satellite markers. A comparison of the two methods indicated a concordance of 92%. Taking into account the chorionicity data, available from the twins’ medical files, in addition to physical similarity led to an increased concordance rate of 96% (Forget-Dubois et al., 2003).

### Japan

The Keio Twin Project includes 1040 pairs of twins and triplets who were recruited between 1998 and 2002 from a population-based twin residential list for the Tokyo area. All twins were native Japanese with age ranging from 14 to 32 years. The twins were invited to participate in the study via mail. Approximately 1000 out of 6000 pairs agreed to participate in the research. A comprehensive questionnaire assessing psychological traits was sent by mail, asking participants to complete it at home and return it. The zygosity of each same-sex twin pair was initially established by a 3-item questionnaire based on physical resemblance (Ooki, Yamada, Asaka, & Hayakawa, 1990). Gene polymorphisms were also examined for 285 pairs. 93.3% of these DNA-based zygosity determinations were in agreement with initial questionnaire-based ones.

### Germany

The Cognitive ability, Self-reported Motivation and School performance Study (CoSMoS) was initiated in 2005. After matching the provided addresses with data found in public telephone directories, 715 families with children twins were contacted by telephone. An additional 1190 households were contacted via mail. Almost two thirds of all personally contacted twins agreed to participate as compared to only 26% of participants contacted by mail. The total number of false positive contacts (people born on the same day and with the same surname who claimed not to be twins) was relatively small (2.4%). To date, the sample has been assessed three times with an interval of two years between testing. Zygosity was established by questionnaires that typically yield accuracies in the magnitude of 95% when compared with zygosity ascertained using DNA markers (Price et al., 2000).

### United States

The Western Reserve Reading Project (WRRP) started in 2002. It is an ongoing longitudinal study that includes 260 pairs of identical (MZ; *n* = 108) and same-sex fraternal (DZ; *n* = 152) twins living in the Greater Cleveland, Columbus, and Cincinnati metropolitan areas, with other families living throughout Ohio and Western Pennsylvania. Twins were recruited into the study when they were in kindergarten or first grade (4.9–7.5), primarily through school nominations (*n *= 293 schools). Schools were asked to forward a packet of information to parents with twins attending kindergarten and not yet completed first grade. The packet included a letter and brochure describing the study and a stamped return postcard addressed to the project offices at the Pennsylvania State University. Additional families were recruited via Ohio State birth records, mothers from twin clubs, and media advertisement. Parents who returned the postcard were then contacted by telephone and, if interested, were sent a 5- to 10-min demographic questionnaire to obtain additional contact information, names and ages of the twins and other children living in the home, parent education, occupation, and ethnicity. Twins were assessed in their homes when they were enrolled into the project and are in the process of annual follow-up home visits. Annual assessments occurred within one month of the anniversary of the previous assessment. Parental permission/informed consent for each assessment was obtained at the time of the home visit. For the majority of twin pairs, DNA was collected via buccal swabs for zygosity determination. In cases where parents did not consent to genotyping (*n* = 76), zygosity was determined using parent questionnaire (Goldsmith, 1991).

### Russia

The Russian School Twin Registry (RSTR) has been established in 2011. The data collection is currently in progress. The main aim of the registry is to contribute to Progress in Education through Gene**-**Environment Studies (PROGRESS). The formation of the registry is ongoing and it is expected that most schools in the Russian Federation (approximately 50,000 schools) will contribute data to the registry. Schools are asked to provide anonymous achievement information on all their enrolled twins (grades 1–11, ages 7–17) and to forward a packet of information to parents of the twins. The parents who give permission for the inclusion of their twins in the RSTR are then contacted directly with request for specific data collection. As part of a large on-going web based assessment, Russian 16-year-old twins complete two questionnaires measuring self-perceived ability and enjoyment in mathematics - validated for the administration in Russia and also completed by TEDS during the assessment at age 16.

## Appendix A.2

### Measures of enjoyment

#### United Kingdom

Three items were used at ages 9 and 12 to measure enjoyment of academic activities by asking participants to answer the following questions using a 5-point Likert scale: “How much do you like” (1) solving number and money problems, (2) doing mathematics in your head, (3) multiplying and dividing – for mathematics; (1) reading, (2) writing, (3) spelling – for English; (1) learning about nature and living things, (2) testing things out to see what they can do, (3) finding out how things work – for science. At age 16, enjoyment in mathematics was measured on a 4-point Likert scale ranging from 1 (strongly disagree) to 4 (strongly agree) using three items: (1) I look forward to my mathematics lessons, (2) I do mathematics because I enjoy it, (3) I am interested in things I learn in mathematics.

#### Canada

Three items were used at ages 10 and 12 to measure enjoyment in mathematics and reading: (1) I like mathematics/reading, (2) Mathematics/Reading interests me a lot, (3) I do mathematics/I read even when I am not obliged to do so. The Likert scale ranged from 1 (Always no) to 5 (Always yes).

#### Japan

Two items were used at ages 10, 11, 12, 13 and 16 to measure mathematics enjoyment: (1) I like mathematics and (2) I study mathematics because I enjoy it. A 6 point-Likert scale was used at all ages.

#### Germany

Three items were used at ages 9, 11 and 13 to measure enjoyment in mathematics and German, by asking the children to answer the following questions on a 5-point scale: “How much do you like” (1) solving number puzzles and text tasks, (2) doing mathematics in your head, (3) multiplying and dividing - for mathematics, and “How much do you like” (1) reading, (2) writing, (3) spelling - for German.

#### United States

Six items were used at age 12 to measure reading enjoyment: (1) I read stories about fantasy and make believe; (2) I like mysteries; (3) I make pictures in my mind when I read; (4) I feel like I make friends with people in good books; (5) I read a lot of adventure stories; and (6) I enjoy a long, involved story or fiction book. The Likert scale ranged from 1 (Very different from me) to 4 (A lot like me).

#### Russia

Russian 16-year-old twins completed two questionnaires that were identical to the ones completed by the TEDS at age 16. The questionnaires were translated and validated for the administration in Russia as part of a large-scale school-based study. Despite the sample size difference, the results for enjoyment were not significantly different between RSTR and TEDS: the group mean difference was not statistically significant, *F*(1, 1738) = .01, *p* > .05, *η*^2 ^= .00; the variance could be equated between the two samples, *F*(1, 1738) = .21, *p* > .05; and the phenotypic correlations between enjoyment and self-perceived ability were also very similar between the TEDS and the RSTR, with overlapping confidence intervals.

### Measures of self-perceived ability

#### United Kingdom

Three items were used at ages 9 and 12 to measure self-perceived ability of academic activities by asking participants to answer the following questions using a 5-point Likert scale: “How good do you think you are at” (1) solving number and money problems, (2) doing mathematics in your head, (3) multiplying and dividing – for mathematics; (1) reading, (2) writing, (3) spelling – for English; (1) learning about nature and living things, (2) testing things out to see what they can do, (3) finding out how things work – for science. At age 16, self-perceived ability in mathematics was measured on a 4-point Likert scale ranging from 1 (Not at all confident) to 4 (Very confident) using eight items: (1) Using a train timetable to work out how long it would take to get from one place to another; (2) Calculating how much cheaper a TV would be after a 30% discount; (3) Calculating how many square meters of tiles you need to cover a floor; (4) Understanding graphs presented in newspaper; (5) Solving an equation like 3*x* + 5 = 17; (6) Finding the actual distance between two places on a map with a 1:10,000 scale; (7) Solving an equation like 2(*x* + 3) = (*x* + 3)(*x* − 3); (8) Calculating the petrol consumption rate of a car.

#### Canada

Three items were used at ages 10 and 12 to measure self-perceived ability in mathematics and reading: (1) I have always done well in mathematics/reading, (2) Mathematics/Reading is easy for me, and (3) I learn things quickly in mathematics/reading. The Likert scale ranged from 1 (Always no) to 5 (Always yes).

#### Japan

Three items were used at ages 10, 11, 12, 13, and 16 to measure self-perceived ability in mathematics: (1) I feel confident in my ability to learn mathematics, (2) I am capable of learning mathematics and (3) I get a good grade in mathematics. A 6 point-Likert scale was used at all ages.

#### Germany

Three items were used at ages 9, 11 and 13 to measure self-perceived ability in mathematics and German, by asking the children to answer the following questions on a 5-point scale: “How good do you think you are at” (1) solving number puzzles and text tasks, (2) doing mathematics in your head, (3) multiplying and dividing – for mathematics; and (1) reading, (2) writing, (3) spelling – for German.

#### United States

At age 12, four items were used to measure self-perceived ability in reading: (1) I know that I will do well in reading next year; (2) I am a good reader; (3) I learn more from reading than most students in the class; and (4) In comparison to my other schools subjects, I am best at reading. The Likert scale ranged from 1 (Very different from me) to 4 (A lot like me). Six items were used to measure self-perceived ability at school studies in general: (1) I am certain I can understand the most difficult material presented in readings; (2) I am confident I can do an excellent job on assignments and tests; (3) I am certain I can master the skills being taught; (4) I learn quickly in most school subjects; (5) I am good at most school subjects; and (6) I can do well in most school subjects. The Likert scale ranged from 1 (Strongly disagree) to 4 (Strongly Agree).

#### Russia

At age 16, the same instrument used for the UK sample was used to measure self-perceived ability. The results were not significantly different between RSTR and TEDS: the group mean difference was not statistically significant, *F*(1, 1728) = .01, *p* > .05, *η*^2 ^= .00; the variance could be equated between the two samples, *F*(1, 1728) = .98, *p* > .05. The phenotypic correlations between enjoyment and self-perceived ability in TEDS and the RSTR were also very similar as these correlations had overlapping confidence intervals.
